# Automated Sidewalk Surface Detection Using Wearable Accelerometry and Deep Learning

**DOI:** 10.3390/s25134228

**Published:** 2025-07-07

**Authors:** Do-Eun Park, Jong-Hoon Youn, Teuk-Seob Song

**Affiliations:** 1 Department of Computer Engineering, Mokwon Uninversity, Daejeon 35349, Republic of Korea; doeun427@mokwon.ac.kr; 2 Department of Computer Science, University of Nebraska, Omaha, NE 68182, USA

**Keywords:** accelerometers, deep neural networks, fast fourier transform, kalman filters

## Abstract

Walking-friendly cities not only promote health and environmental benefits but also play crucial roles in urban development and local economic revitalization. Typically, pedestrian interviews and surveys are used to evaluate walkability. However, these methods can be costly to implement at scale, as they demand considerable time and resources. To address the limitations in current methods for evaluating pedestrian pathways, we propose a novel approach utilizing wearable sensors and deep learning. This new method provides benefits in terms of efficiency and cost-effectiveness while ensuring a more objective and consistent evaluation of sidewalk surfaces. In the proposed method, we used wearable accelerometers to capture participants’ acceleration along the vertical (*V*), anterior-posterior (AP), and medio-lateral (ML) axes. This data is then transformed into the frequency domain using Fast Fourier Transform (FFT), a Kalman filter, a low-pass filter, and a moving average filter. A deep learning model is subsequently utilized to classify the conditions of the sidewalk surfaces using this transformed data. The experimental results indicate that the proposed model achieves a notable accuracy rate of 95.17%.

## 1. Introduction

Walkability refers to the ability of individuals to safely and comfortably walk to essential services, amenities, and destinations within a reasonable distance. It is a key factor in urban planning and transportation, directly impacting public health, environmental sustainability, and overall quality of life [1,2,3,4,5]. Walkability studies provide valuable insights that help city planners design safer and more accessible sidewalks, crosswalks, and public spaces. Data-driven assessments can reveal disparities in walkability across neighborhoods, guiding efforts toward more inclusive and equitable urban design. Moreover, enhancing sidewalk conditions can reduce the risk of falls and promote daily physical activity, which is essential for preventing obesity, cardiovascular disease, diabetes, and mental health challenges.

Recognizing the importance of walkability, city leaders and policymakers worldwide are making concerted efforts to enhance pedestrian-friendly environments by improving infrastructure, reducing vehicular dependence, and creating safer, more accessible pathways for pedestrians. One of the primary methods used to assess walkability is pedestrian interviews and surveys, which gather insights into how people perceive and experience sidewalks and pedestrian spaces. These surveys serve as a representative evaluation tool that takes into account subjective aspects such as perceived safety, convenience, and comfort [4,6,7,8,9,10]. However, while these responses provide valuable qualitative data, they are inherently prone to biases and individual subjectivity. Factors such as personal preferences, prior experiences, and environmental conditions at the time of the survey can influence the results, leading to inconsistencies and a lack of expert-driven assessment.

To ensure a more structured and regulation-compliant evaluation of pedestrian spaces, government agencies often rely on trained experts to conduct on-site inspections. These experts evaluate sidewalk conditions, verify compliance with accessibility standards, and assess the overall adequacy of pedestrian infrastructure based on pre-established regulatory frameworks. While expert evaluations provide more objective insights, they come with significant drawbacks. Traditional on-site inspections are time-consuming, labor-intensive, and require substantial financial resources. The logistical challenges of deploying experts to assess large urban areas make these methods inefficient, especially in rapidly growing cities with evolving pedestrian needs. Furthermore, conventional assessment methods often fail to adequately capture the dynamic and contextual elements that influence walkability. Pedestrian behavior, real-time environmental changes, and situational factors, such as weather conditions, traffic patterns, and urban design intricacies, play a crucial role in shaping the pedestrian experience [7,11,12,13,14,15,16,17,18,19,20]. Relying solely on static assessments may overlook these vital aspects, leading to incomplete or outdated evaluations. As a result, there is a growing need for innovative and data-driven approaches that can provide more comprehensive, real-time insights into walkability while optimizing resource allocation for urban planners and decision-makers.

In this paper, we propose an automated method for recognizing sidewalk types using wearable sensors that take pedestrian behavior into account. Wearable sensors can be used on pedestrians to monitor their physiological responses, allowing for an analysis of how the surrounding environment affects these responses. Previous research has demonstrated that various sidewalk characteristics or defects cause changes in human responses [21,22]. Our proposed method recognizes sidewalk surface types by extracting features from time-domain data, including average, standard deviation, and Single Value Magnitude (SVM). We employed 15 features derived from SVM, which were further refined using Kalman filters, moving averages, and low-pass filters. In addition, we incorporated frequency-domain features, such as those obtained through Fast Fourier Transform (FFT), to enhance surface type classification [23].

The main contributions of the study are summarized as follows:

- A novel method is proposed that integrates deep learning with Kalman filtering and Fast Fourier Transform (FFT), leveraging foundational techniques in signal processing to classify various sidewalk surface types. This approach holds promise for broader applications in human behavior recognition and activity classification.
- Unlike previous studies [8,9], which focused solely on distinguishing between normal and abnormal surface conditions, the proposed method effectively classifies five distinct sidewalk surface types, achieving higher recognition accuracy.
- By utilizing a relatively simple deep learning architecture [14,17], the proposed approach effectively classifies multiple sidewalk surface types while maintaining computational efficiency. Details of the model structure are provided in Table 1.
- The study also investigates optimal sensor placement for sidewalk surface detection using wearable devices. Recognition accuracy is evaluated across single-, dual-, and tri-sensor configurations (see Table 2). The configuration combining hip and ankle sensors yielded the highest accuracy.

This paper is organized as follows. Section 2 reviews previous studies on walkability and sidewalk surface recognition. Section 3 presents the feature extraction from accelerometer data and the deep learning modeling for surface recognition. Section 4 describes the experimental results, and Section 5 provides the conclusion and directions for future research.

## 2. Related Works

This section presents a comprehensive overview of existing sidewalk evaluation methods. Section 2.1 covers traditional approaches involving on-site inspections, checklists, and expert assessments and examines their limitations, including subjectivity, time and labor intensity, and spatial constraints. Section 2.2 explores recent technological advancements, highlighting machine learning and deep learning-based methods. In particular, it focuses on approaches that leverage human activity recognition techniques to automatically detect and interpret human movements and behaviors. The discussion emphasizes how these modern methods enhance the efficiency and objectivity of sidewalk evaluation, marking a shift from conventional practices.

### 2.1. Traditional Approaches for Sidewalk Assessment

As sidewalk assessment is essential for evaluating walkability, numerous methods have been developed to assess sidewalk conditions. Traditionally, pedestrian surveys have been among the most widely used approaches for identifying sidewalk defects [21]. These surveys typically involve pedestrians or inspectors physically walking through an area to detect issues. However, this method relies heavily on the subjective judgment of the observer, limiting its accuracy and making it unsuitable for quantitatively analyzing the characteristics or severity of defects [8,21]. Additionally, it is time- and labor-intensive, leading to high costs and limited coverage, which reduces overall efficiency [8]. Government agencies also commonly conduct on-site inspections using trained personnel to assess compliance with regulations and evaluate sidewalk conditions [24]. While these inspections offer more systematic evaluations by skilled professionals, they are similarly constrained by high labor costs and extended inspection durations, making them impractical for large-scale implementation [9,24].

To address the limitations of traditional approaches, researchers have proposed various methods aimed at evaluating sidewalk conditions more efficiently and objectively. For instance, Sousa et al. developed a technique based on on-site measurements, enabling a degree of quantitative analysis [25]. Another study introduced a method for calculating the Pavement Condition Index (PCI) using survey data tailored specifically for sidewalk assessment. PCI provides a quantitative measure of pavement and sidewalk quality, serving as a valuable standard for prioritizing repairs and allocating maintenance budgets [22].

Recent research on sidewalk quality assessment integrates objective physical attributes with subjective pedestrian experiences. A study in Tegal City revealed that 72% of pedestrians experienced discomfort due to uneven surfaces, inadequate width, and poor maintenance, highlighting the need for infrastructure improvements [26]. The SWAUR framework offers a comprehensive approach by combining observational audits with pedestrian perception surveys to evaluate walkability at both sidewalk and neighborhood levels [27].

Despite these advancements, several challenges remain. Many of these methods still depend on on-site inspections, making them labor-intensive and requiring human involvement, an obstacle to full automation. Moreover, their applicability is often limited to specific geographic regions or contexts, reducing their scalability and effectiveness in large, densely populated urban areas [9]. Consequently, the persistent limitations of traditional methods underscore the need for sensor-based approaches to sidewalk condition assessment.

### 2.2. Advanced Approaches for Sidewalk Assessment

Sensor-based and machine learning approaches are increasingly being adopted for sidewalk evaluation, addressing the limitations of traditional survey-based methods, which are often costly, labor-intensive, and subject to observer bias. In contrast, sensor-based techniques offer scalable, objective, and data-driven assessments of sidewalk conditions. For example, accelerometer data combined with a Support Vector Machine (SVM) classifier was used to identify structural defects, such as horizontal and vertical cracks and surface holes [21]. Building upon this approach, Kim et al. [24] proposed a method for distinguishing between unobstructed sidewalks and those compromised by fallen leaves or physical barriers, utilizing both accelerometer and heart rate sensor data to enhance classification accuracy. In a separate study, Takahashi et al. [28] used smartphone-based accelerometers to classify road gradient conditions—flat, uphill, and downhill—during cycling, with the device placed in different locations such as a pants pocket, chest pocket, and bicycle basket.

Further advancing this line of work, Landis et al. [7] investigated sidewalk surface classification using Inertial Measurement Units (IMUs) placed on the head, waist, and ankle. Surface types were categorized as normal or abnormal (e.g., grass-covered paths, debris-laden areas, and uneven surfaces), and multiple machine learning algorithms—including SVM, Random Forest, and Logistic Regression—were evaluated in terms of classification performance relative to sensor placement. More recently, the SideSeeing project introduced a publicly available multimodal dataset comprising IMU, GPS, and synchronized video data, designed for sidewalk accessibility analysis near hospitals in Brazil and the United States [29]. This dataset supports advanced analytics and model training for inclusive pedestrian infrastructure. Additionally, the findings reported in Sensors revealed that certain commonly used sidewalk materials fail to meet safety standards under wet conditions, highlighting the importance of material-level assessments in ensuring pedestrian safety [30].

While sensor-based walkability assessment offers the advantage of collecting objective, high-resolution, and continuous data on pedestrian movement and environmental conditions, several challenges remain. These include the need to manage and interpret large, complex datasets, which often require advanced analytical and machine learning techniques. Additionally, continuous data collection raises important privacy concerns, particularly when wearable or location-tracking sensors are involved. The accuracy and generalizability of sensor-based systems can also be influenced by factors such as sensor placement, device calibration, environmental variability, and individual differences in gait patterns [31].

With advances in deep learning, numerous studies have leveraged these techniques for road and sidewalk condition recognition. In [32], a method was developed to detect environmental barriers to wheelchair navigation—such as stairs, steep slopes, and doorways—using accelerometer data collected from smartphones. Time-domain features, including mean, standard deviation, and inter-axis correlations (x–y, x–z, y–z), were extracted and used to train a Deep Neural Network (DNN). Building upon earlier work in [8], the authors in [9] employed ankle-mounted inertial sensors and a Long Short-Term Memory (LSTM) network to classify sidewalk surfaces into five types, further categorizing them as normal or abnormal based on their walkability characteristics. Additionally, [33] proposed a smartphone-based approach for recognizing six different types of road surfaces by extracting both time- and frequency-domain features—such as maximum amplitude and standard deviation across each axis—and evaluated model performance using Convolutional Neural Networks (CNNs) and VGG16 architectures.

## 3. Classification of Sidewalk Surface Types Using Deep Learning and Signal Processing

**Figure 1:**
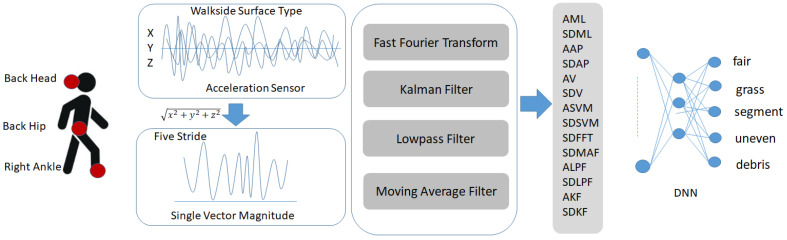
Framework of the proposed system.

This section elaborates on the data analysis process used to recognize various sidewalk surface types. The process begins by computing the single vector magnitude (SVM) from the three-axis acceleration data (x, y, and z) collected by wearable sensors. SVM provides a consolidated measure of overall motion intensity, independent of direction. After calculating the SVM, several signal processing techniques are applied to extract informative features. These include FFT for frequency-domain analysis, along with Kalman filtering, low-pass filtering, and moving average filtering, to suppress noise and enhance signal quality. The resulting combination of time-domain and frequency-domain features serves as input to a Deep Neural Network (DNN), which classifies the sidewalk surface into one of five predefined categories. Figure 1 depicts the overall structure of our proposed system.

### 3.1. Data Collection

A total of 12 healthy subjects (8 males and 4 females ) were recruited to participate in the study. The subjects wore three tri-axial accelerometers at different locations on the body. It is anticipated that the head-mounted sensor will capture key characteristics of head movement associated with walking on unstable or obstructed sidewalks, including active head scanning, as well as downward and lateral gaze shifts used to monitor foot placement and safely navigate around environmental obstacles [34]. The hip sensor, positioned near the body’s center of mass, is expected to provide a stable and representative measure of whole-body movement dynamics during locomotion [35]. In parallel, foot-mounted sensors—due to their direct interaction with the walking surface—will yield high-resolution, event-level data for gait analysis, including precise detection of heel-strike, toe-off, and other temporal-spatial gait parameters [36]. Together, these sensor modalities offer a comprehensive view of locomotor adaptation in response to challenging walking environments.

**Figure 2:**
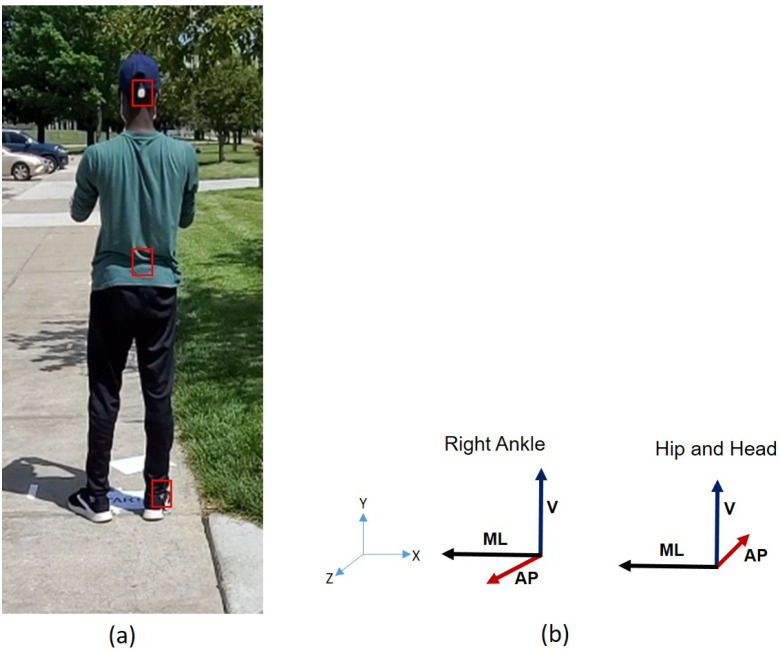
(**a**) Placement of sensors at three locations on a subject. (**b**) Accelerometers’ directions.

As shown in Figure 2, the head accelerometer was mounted on the back of a cap, and the hip accelerometer was affixed to the back of the hip. The right ankle accelerometer was mounted on the outer side of a subject’s shoe. The linear accelerations of the body were measured using Mbient sensors [37]. These tri-axial accelerometers were configured to sample data at 100 Hz in the range of ±4 g. The acceleration data were synchronously and wirelessly transferred to a mobile phone through a Bluetooth network. The data were then transferred to a notebook computer for offline analysis.

**Figure 3:**
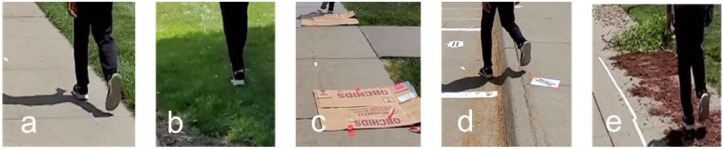
Good and typical irregular walking surfaces: (**a**) Good, well-paved segment (Type 0); (**b**) grass-covered segment (Type 1); (**c**) obstacles with physical obstructions (Type 2); (**d**) uneven surface segment (Type 3); and (**e**) debris-covered segment (Type 4).

The experiment was designed to approximate real-world walking conditions. A section of smooth, level, and well-maintained pavement located at the Peter Kiewit Institute, University of Nebraska at Omaha, was selected as the experimental site. The walking path was clearly marked with designated starting and ending points. To simulate a sidewalk environment, masking tape was applied to the ground to define the boundaries. Within this controlled pathway, four distinct irregular surface segments were introduced to replicate commonly encountered sidewalk conditions: a grass-covered segment (Type 1), obstacles with physical obstructions (Type 2), an uneven surface (Type 3), and a debris-covered segment (Type 4). These variations were chosen to reflect typical challenges pedestrians may face in urban settings. Figure 3 provides an overview of the experimental layout, including both the regular and irregular walking surface segments.

Participants were instructed to walk along the designated path at their normal, self-selected walking speed. Upon reaching the endpoint, each participant was asked to pause for 10 s before retracing the path in the opposite direction, returning to the starting point. Throughout the experiment, researchers followed behind the participants and recorded video footage to assist with subsequent data labeling and validation. The total duration of the task varied between participants, as walking speed was not standardized in order to reflect naturalistic gait patterns typical of real-world conditions. Each participant walked at a moderate pace (approximately 3 m per hour) for a duration of 10 min, resulting in a total round-trip distance of approximately 0.5 m.

For the purpose of data analysis, we generated a total of 600 simulation datasets, each derived from acceleration sensor data recorded while a pedestrian was walking on different types of sidewalk surfaces. These sidewalk surfaces were classified into five distinct categories: fair, grass, obstacles, uneven, and debris. Each category represents a different walking condition, affecting the pedestrian’s movement and the sensor readings accordingly.

To ensure a diverse and balanced dataset, we constructed 10 independent datasets for each sidewalk surface condition. As there are five different surface types, this resulted in a total of 600 datasets. Each dataset was extracted from acceleration sensor readings collected during pedestrian movement and consisted of 515 data points, which corresponded to a walking sequence of five consecutive steps. The selection of this window size was based on an analysis of step patterns and sensor signal characteristics to effectively capture the variations in movement across different surfaces.

**Figure 4:**
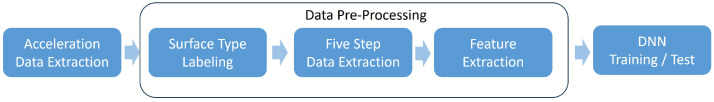
Workflow for classifying sidewalk surface types using deep learning.

**Figure 5:**
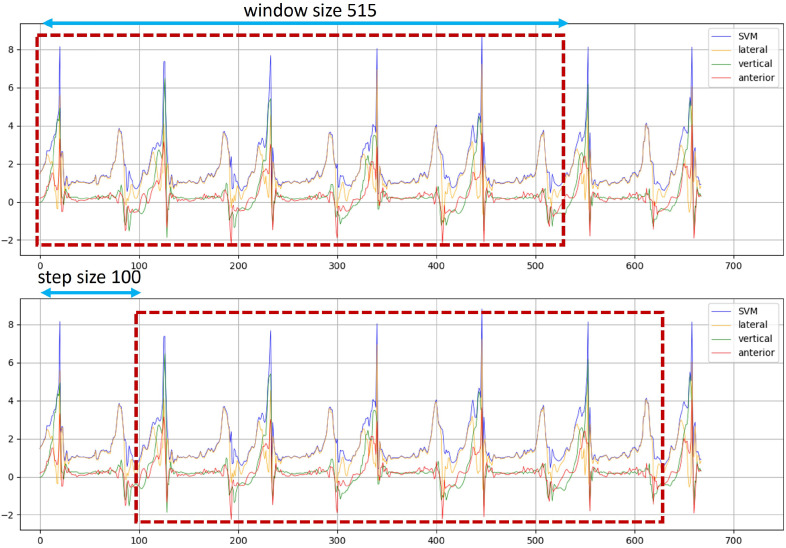
Constructed datasets using the sliding window method. The red dashed boxes indicate the window size.

The datasets were created using a sliding window approach, a common method in time-series data analysis. Specifically, the window size was set to 515 data points, ensuring that each dataset covered a walking segment of five steps. To facilitate continuous data extraction while preserving temporal dependencies, a step size of 100 was applied. This means that instead of extracting entirely independent windows, each subsequent dataset partially overlapped with the previous one, allowing for a richer and more detailed representation of walking dynamics across different sidewalk surfaces. By employing this sliding window method, we were able to generate a comprehensive dataset that captures the variations in acceleration signals influenced by different sidewalk conditions. Figure 4 shows the complete data analysis workflow, beginning with the data collection phase. This data construction process is illustrated in Figure 5, providing a visual representation of how the windows were selected and how the step size was applied during data extraction.

### 3.2. Feature Extraction Using FFT, Kalman, Low Pass, and Moving Average Filters

The three-axis accelerometer measures the acceleration magnitude in the vertical (*V*), antero-posterior (AP), and medio-lateral (ML) directions. By calculating the SVM of the three acceleration magnitudes, as shown in Equation (1), we can determine the overall acceleration acting on the object, which is commonly used in acceleration-based research.

(1) $$SVM_{k}=\sqrt{{ML_{k}}^{2}+{AP_{k}}^{2}+{V_{k}}^{2}}$$

where the k=0,1,…,514.

The single vector magnitude (or the magnitude of the acceleration vector) is a way to combine the acceleration values from all three axes into a single scalar value that represents the overall acceleration, regardless of direction. It gives the total acceleration experienced by the object or device in 3D space. Time-domain features, including the mean and standard deviation, were extracted based on the SVM. Additionally, the mean and standard deviation for each directional axis were also computed and utilized as feature inputs.

From a signal processing perspective, FFT is a mathematical tool used to analyze and decompose a signal into its frequency components. In this context, a signal is typically a time-domain function, and the Fourier transform converts this time-domain signal into a frequency-domain representation. This transformation reveals the presence and magnitude of various frequencies within the original signal, which is crucial for understanding, filtering, or modifying the signal. The output of the Fourier transform is a complex-valued function, where the magnitude represents the amplitude of each frequency component, and the phase provides information about the timing or phase shift of these components [38,39]. In the study, we analyzed the frequency components of the SVM, given as (2)

(2) $$X_{k}=\sum_{n=0}^{N-1}SVM_{n}\exp\left\{-j2\pi \frac{k}{N}n\right\},\;k=0,1,\ldots ,N-1$$

where X(k) represents the frequency-domain representation of the signal at frequency index *k*, and *N* is the total number of acceleration samples within a window size of 515.

A Kalman filter is an algorithm used to estimate the state of a system over time, particularly when the system is subject to uncertainty or noise. It is widely used in control systems, signal processing, and robotics for tracking or filtering signals. The Kalman filter provides an optimal estimation of a system’s state by combining measurements from sensors with a mathematical model of the system, taking into account both the noise in the measurements and the uncertainty in the model. The initial input value of the Kalman filter algorithm is set to $SVM_{0}$.

(3) $${\hat{x}}_{0}=SVM_{0}$$

The Equations (5)–(7) describe the algorithm for applying a standard Kalman filter. ${\hat{x}}_{k}$ represents the estimated value of the Kalman filter. For k=1,2,…,514,

(4) $$\begin{array}{ccc} {\hat{x}}_{k}^{-} & = & {\hat{x}}_{k-1}, \end{array}$$

(5) $$\begin{array}{ccc} P_{k}^{-} & = & P_{k-1}+Q, \end{array}$$

(6) $$\begin{array}{ccc} K_{k} & = & \frac{P_{k}^{-}}{P_{k}^{-}+R}, \end{array}$$

(7) $$\begin{array}{ccc} {\hat{x}}_{k} & = & {\hat{x}}_{k}^{-}+K_{k}\left(z_{k}-{\hat{x}}_{k}^{-}\right). \end{array}$$

It is well known that the Kalman filter is applied recursively; therefore, after updating Equation (8), the process is repeated at each time step.

(8) $$P_{k}=\left(1-K_{k}\right)P_{k}^{-}$$

To compute the Kalman filter, we set the process noise covariance to $Q=10^{-5}$ and R=0.01 and initialized the error covariance matrix *P* as a zero matrix of size 514.

A low-pass filter is an electronic or mathematical filter that allows signals with frequencies lower than a certain cutoff frequency to pass through while attenuating (reducing) the amplitude of signals with frequencies higher than the cutoff frequency. Essentially, it “passes” low-frequency signals and blocks or reduces high-frequency signals, making it useful for noise reduction or signal smoothing [40]. We obtain the low-pass filter from SVM as (9).

(9) $$LPF_{k}=\alpha LPF_{k-1}+\left(1-\alpha \right)SVM_{k},\;0\leq \alpha \leq 1$$

where k=1,…,514.

**Figure 6:**
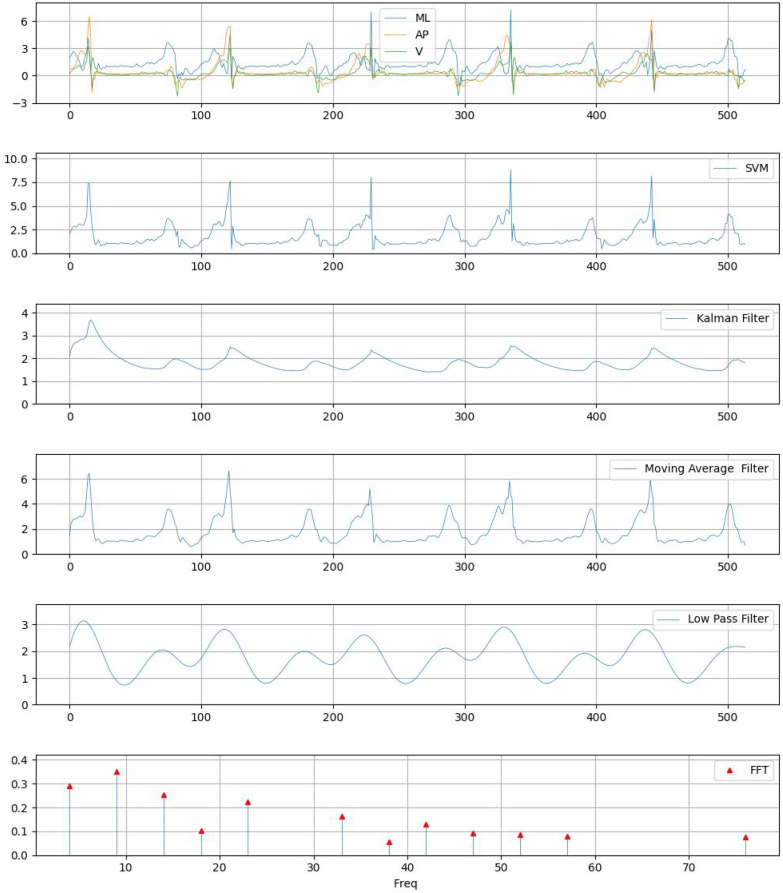
Example graph of the acceleration values of each axis, SVM, low-pass filter, Kalman filter, moving average filter, and FFT.

A moving average filter is a simple digital filter used to smooth or reduce noise in time series or signal data by averaging a fixed number of consecutive data points, called the window size. The window size determines how many data points are included in each average calculation; a smaller window size preserves more details but offers less smoothing, whereas a larger window size provides greater smoothing but may diminish responsiveness to short-term changes. This process helps eliminate high-frequency noise while preserving the general trend of the signal [40]. For an example of applying these filters to raw signals, please see Figure 6. We calculated the moving average filter using SVM, as per (10) and (11). For k=0,1,…,514,

(10) $$MAF_{k}=\frac{1}{k}\sum_{i=n-k+1}^{n}SVM_{i},$$

(11) $$MAF_{k+1}=MAF_{k}+\frac{1}{k}\left(SVM_{n+1}-SVM_{n-k+1}\right).$$

### 3.3. Structure of Deep Neural Network

The deep learning architecture employed for recognizing sidewalk surface types was based on a DNN. Table 3 presents the features extracted for recognizing sidewalk surface conditions. A total of 15 features were used, including 8 time-domain features, 6 filter-based features, and 1 frequency-domain feature. As shown in Table 1, the DNN consists of an input layer, two hidden layers, and one output layer. As for the activation function, ReLU was used for the hidden layer, and the Sigmoid function was used for the output layer. The neural network included two hidden layers with 1000 and 800 nodes, respectively. The output layer consisted of five nodes, each representing one of the five surface types to be recognized. Adam and CrossEntropy functions were used as optimization and loss functions, respectively. The learning rate was set to 0.001.

## 4. Experimental Results

Twelve participants, labeled A through L, took part in the experiment. We conducted experiments using a 10-fold cross-validation across five different sidewalk surface types. Specifically, we created 10 experimental datasets for each surface type, numbered 0 through 9. Each experiment was performed by training on nine datasets while excluding one for testing. Experiments #1, #2, and #3 were conducted using a single sensor attached to the head, waist, and ankle, respectively. Experiments #4, #5, and #6 involved two sensors in different combinations: head and waist, waist and ankle, and head and ankle. Lastly, Experiment #7 utilized all three sensors simultaneously.

In this study, we conducted two types of experiments to analyze how the composition of features affects classification performance. The first experiment utilized only 14 features extracted from the time domain, excluding any frequency-domain information. In contrast, the second experiment employed a total of 15 features, which included the same 14 time-domain features, along with an additional frequency-domain feature derived using FFT. The results of each experiment are summarized in Table 2 and Table 4. When comparing the experimental results, we observed that, despite both experiments being conducted under similar conditions, the inclusion of the FFT feature consistently led to higher classification accuracy. This suggests that frequency-domain information can complement time-domain features by capturing signal patterns or characteristics that are otherwise difficult to identify. Therefore, we confirmed through experimentation that incorporating the FFT feature contributes to improving the model’s performance. Table 4 presents the experimental results using 14 features, excluding the frequency domain feature SDFFT.

Table 2 presents the experimental results obtained using all features, including the frequency domain feature SDFFT. In single-sensor experiments (#1–#3), the hip sensor recorded the lowest accuracy at 78.0%, while the ankle sensor achieved the highest accuracy at 88.17%. Among the two-sensor setups (#4–#6), the head and hip combination had the lowest accuracy at 84.67%, whereas the hip and ankle combination in Experiment #6 yielded the highest accuracy. Although Experiment #7, which incorporated all three sensors, was expected to provide the highest accuracy, Experiment #6 slightly outperformed it. However, Experiment #7 demonstrated the lowest standard deviation, indicating more consistent results across trials.

**Figure 7:**
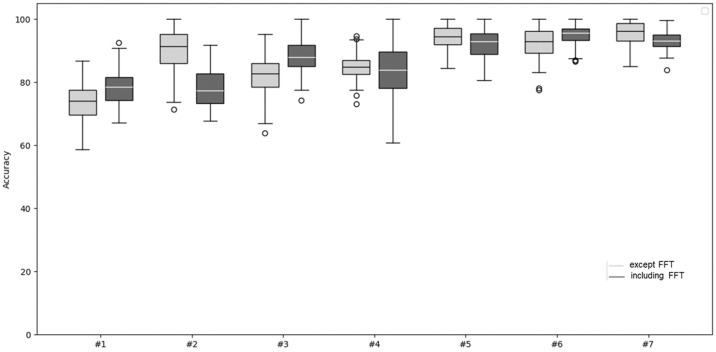
Comparison of the two experiments, without FFT and with FFT.

Figure 7 shows box plots derived from the data in Table 2 and Table 4. As shown in the box plots, Experiment #6, which includes the FFT feature, demonstrates the best overall performance among all the experiments. This is evident from its consistently higher median values, narrower interquartile range, and fewer outliers, indicating both superior accuracy and stability. The incorporation of the FFT feature appears to have significantly contributed to this improved performance.

Table 5 shows the accuracy of Experiment #6. While the experimental participants E, H, and L achieved 100% accuracy, the model correctly identified only 44 out of 50 sidewalk surface types in test case I, showing a recognition rate of 88%.

**Figure 8:**
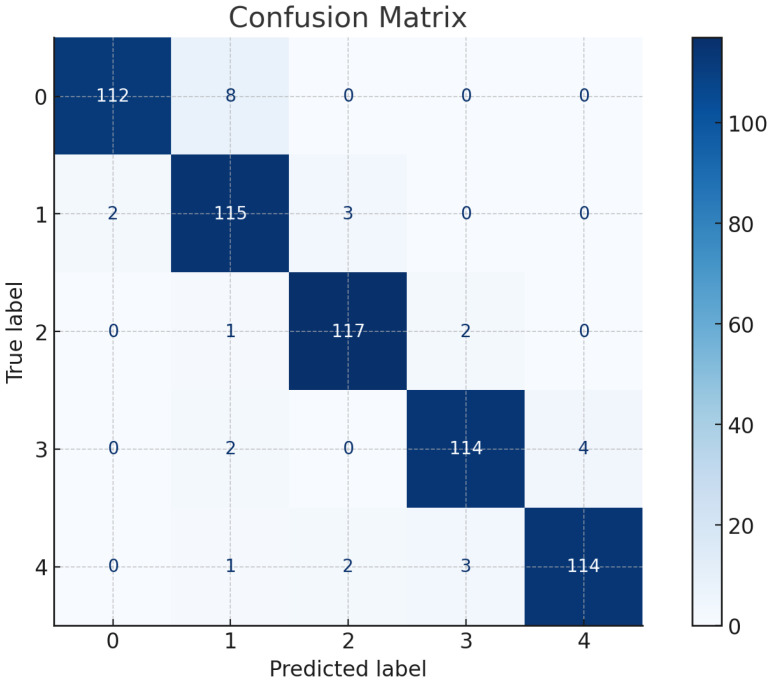
Confusion matrix of sidewalk surface types.

Figure 8 shows the confusion matrix for each participant in Experiment #6. It can be observed that the sidewalk surfaces of a grass-covered segment (Type 1) and obstacles with physical obstructions (Type 2) achieved the highest accuracy.

The F1 score is a widely used evaluation metric in classification problems, which is defined as Equations (12) and (13):

(12) $$F1=2\cdot \frac{\mathrm{Precision}\cdot \mathrm{Recall}}{\mathrm{Precision}+\mathrm{Recall}}.$$

Precision and recall are defined as

(13) $$\mathrm{Precision}=\frac{TP}{TP+FP}\;\mathrm{and}\;\mathrm{Recall}=\frac{TP}{TP+FN}$$

where TP is True Positive, FP is False Positive, and FN is False Negative. Based on the results summarized in Figure 8, the calculated F1 score reaches 0.972, indicating a high level of balance between precision and recall in the model’s performance.

**Figure 9:**
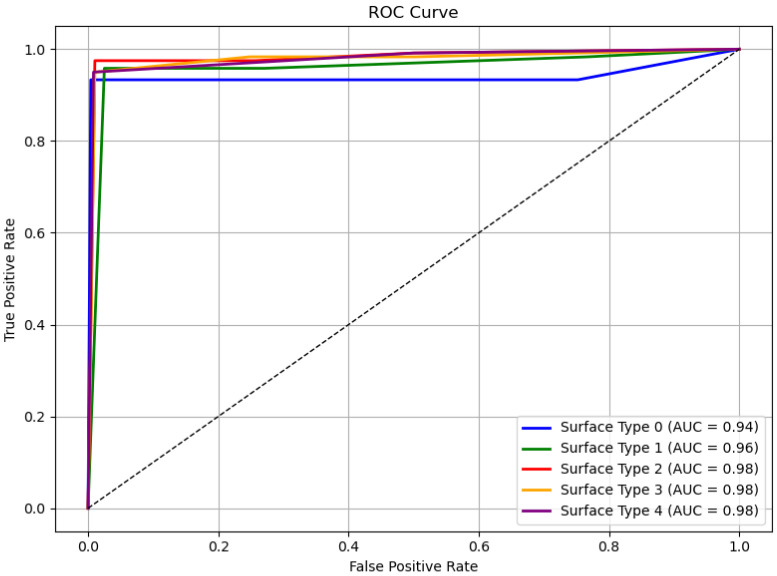
A graph of the ROC curves.

Figure 9 presents the ROC curve based on the results from Figure 8, demonstrating the model’s excellent classification performance across all sidewalk surface types [41].

**Figure 10:**
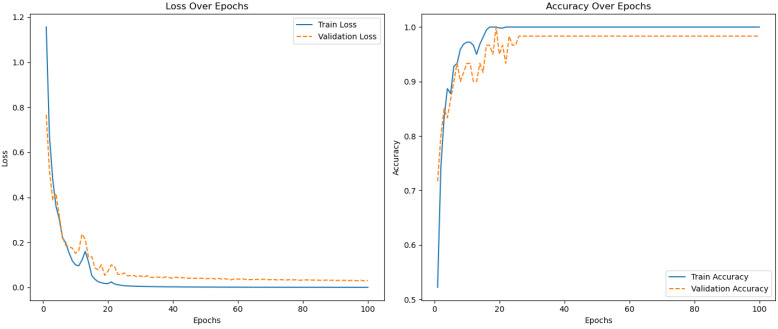
An example of training error and accuracy.

Figure 10 shows the error and accuracy graph for Experiment #6, which achieved the highest accuracy.

Table 6 presents a comparison between previous studies and the experimental results of this study. Ng [8] proposed a machine learning-based approach to classify normal and abnormal road surface conditions. By applying a Support Vector Machine to five surface types, they achieved a maximum recognition accuracy of 87%. Subsequently, Ng [9] adopted a deep learning-based approach using LSTM, which resulted in an improved accuracy of 88% for the same classification task. Meanwhile, Miyata [32] and Kobayashi [33] combined time-domain and frequency-domain features, achieving recognition accuracies of 85% and 71.44%, respectively. In comparison, the method proposed in this study achieved a recognition accuracy of 95.17% across five surface types, demonstrating superior performance over the existing approaches.

## 5. Conclusions

Assessing walkability is an essential aspect of urban planning and public health research, as it provides insights into pedestrian accessibility, safety, and overall walking experience. Traditionally, pedestrian interviews and surveys have been widely used for this purpose. However, these methods can be both costly and resource-intensive, particularly when applied at a large scale. Conducting large-scale surveys requires substantial human effort, time, and financial resources, making it challenging to implement in diverse urban environments.

To address these limitations, this paper proposes a novel approach to walkability assessment through the use of wearable accelerometers. Specifically, we introduce a method for automatically recognizing sidewalk surface conditions using a body-worn sensor. By leveraging acceleration data collected from wearable devices, our approach enables real-time and cost-effective evaluation of pedestrian pathways without the need for extensive manual surveys. For the classification of sidewalk surface types, we employed FFT and Kalman filter techniques, both of which are widely utilized in signal processing. FFT is used to analyze the frequency components of the accelerometer signals, helping to identify distinctive patterns associated with different walking surfaces. Meanwhile, the Kalman filter is applied to enhance the accuracy of surface recognition by reducing noise and improving signal stability.

To further enhance classification performance, we incorporated deep learning techniques into our analysis. The experimental results show that the inclusion of the FFT-derived feature significantly contributes to achieving high accuracy. The suggested method offers improved accuracy in distinguishing various sidewalk surface conditions. These findings highlight the potential of wearable accelerometers as a practical tool for automated walkability assessment.

The findings underscore the promise of this approach; however, its application within urban design—particularly in fostering environments that are accessible and accommodating to children and individuals with disabilities—requires further exploration. Furthermore, we plan to investigate an end-to-end deep learning approach to more precisely recognize sidewalk surface conditions. Specifically, we aim to design a model that directly learns features from raw accelerometer data, eliminating the need for manual feature extraction. To achieve this, we will implement a hybrid model that combines LSTM networks for capturing temporal patterns and CNN for extracting spatial features.

## Figures and Tables

**Table 1:** Summary of the proposed DNN model.

Layer	Output Shape	Parameters
Linear	[−1, 1000]	16,000
ReLU	[−1, 1000]	0
Linear	[−1, 800]	800,800
ReLU	[−1, 800]	0
Linear	[−1, 5]	4005
Sigmoid	[−1, 5]	0

**Table 2:** Comparative accuracy of experimental results with the FFT feature.

No.	#1	#2	#3	#4	#5	#6	#7
0	73.33	70.00	80.00	76.67	83.33	93.33	91.67
1	80.00	78.33	90.00	91.67	100.00	96.67	98.33
2	81.67	78.33	93.33	88.33	93.33	100.00	93.33
3	81.67	71.67	91.67	86.67	91.67	93.33	95.00
4	83.33	78.33	91.67	93.33	93.33	95.00	93.33
5	86.67	88.33	88.33	93.33	95.00	100.00	96.67
6	86.67	85.00	90.00	90.00	95.00	96.67	93.33
7	71.67	76.67	91.67	80.00	96.67	95.00	91.67
8	76.67	73.33	90.00	78.33	88.33	93.33	90.00
9	71.67	80.00	75.00	68.33	88.33	88.33	90.00
avg	79.34	78.00	88.17	84.67	92.50	95.17	93.33
std	5.44	5.36	5.60	7.99	4.55	3.29	2.58

Exper. #1 uses head, Exper. #2 uses hip, Exper. #3 uses ankle, Exper. #4 uses head + hip, Exper. #5 uses head + ankle, Exper. #6 uses hip + ankle, and Exper. #7 uses head + hip + ankle.

**Table 3:** Description of the 15 selected features.

Category	Feture	Description
Time Domain	AML	average of ML-axis for 515 acceleration value
	SDML	standard deviation of ML-axis for 515 acceleration value
	AAP	average of AP-axis for 515 acceleration value
	SDAP	standard deviation of AP-axis for 515 acceleration value
	AV	average of *V*-axis for 515 acceleration value
	SDV	standard deviation of V-axis for 515 acceleration value
	ASVM	average of SVM for 515 acceleration value
	SDSVM	standard deviation of SVM for 515 acceleration value
Filter Domain	AMAF	average of moving average filter for SVM 515 acceleration value
	SDMAF	standard deviation of moving average filter for SVM 515 acceleration value
	ALPF	average of low pass filter for SVM 515 acceleration value
	SDLPF	standard deviation of low pass filter for SVM515 acceleration value
	AKF	average of Kalman filter for SVM 515 acceleration value
	SDKF	standard deviation of Kalman filter for SVM 515 acceleration value
Frequency Domain	SDFFT	standard deviation of FFT for SVM 515 acceleration value

**Table 4:** Comparative accuracy of experimental results with the FFT feature excluded.

No.	#1	#2	#3	#4	#5	#6	#7
0	56.67	78.33	78.33	73.33	88.33	91.67	91.67
1	83.33	91.67	95.00	90.00	98.33	95.00	95.00
2	76.67	91.67	95.00	85.00	93.33	98.33	96.67
3	78.33	88.33	93.33	90.00	93.33	93.33	95.00
4	78.33	95.00	86.67	85.00	95.00	96.67	93.33
5	80.00	91.67	86.67	88.33	93.33	90.00	95.00
6	75.00	93.33	90.00	80.00	95.00	95.00	96.67
7	68.33	78.33	81.67	80.00	90.00	95.00	93.33
8	75.00	80.00	85.00	83.33	86.67	93.33	90.00
9	65.00	83.33	75.00	76.67	88.33	85.00	88.33
avg	73.67	87.17	86.67	83.17	92.17	93.33	93.50
std	7.63	6.20	6.54	5.35	3.50	3.57	2.63

Exper. #1 uses head, Exper. #2 uses hip, Exper. #3 uses ankle, Exper. #4 uses head + hip, Exper. #5 uses head + ankle, Exper. #6 uses hip + ankle, and Exper. #7 uses head + hip + ankle.

**Table 5:** Number of correct recognitions by each person in experiment #6.

Sub.	A	B	C	D	E	F	G	H	I	J	K	L
Num. Corr.	48	48	49	48	50	45	47	50	44	47	46	50
Rate Corr.	96%	96%	98%	96%	100%	90%	94%	100%	88%	94%	92%	100%

**Table 6:** Comparison of simulation results.

Ref.	Features	Model	Recognition Categories	Acc.
Ng et al. [8]	20 time-domain features	Support Vector Machine	well-paved, grass-covered, obstacles with physical obstructions, uneven surface, debris-covered	87%
Ng et al. [9]	20 time-domain features	LSTM	well-paved, grass-covered, obstacles with physical obstructions, uneven surface, debris-covered	88%
Miyata et al. [32]	9 time-domain features, 27 frequency-domain features	Support Vector Machine	flat, up/down stairs, up/down step, low/high slope, swinging door	85%
Kobayasi et al. [33]	20 time-domain features, 10 frequency-domain features	VGG16	asphalt, gravel, lawn, grass, sand, mat	71.44%
Proposed	8 time-domain features, 6 filter-domain features, 1 frequency-domain feature	DNN	well-paved, grass-covered, obstacles with physical obstructions, uneven surface, debris-covered	95.17%

## Data Availability

The data presented in this study are contained within the article, and additional information is available upon request from the corresponding author.

## References

[1] Ro R.H. (2009). Walkability: What is it?. J. Urban. Int. Res. Placemaking Urban Sustain..

[2] Ball K., Bauman A., Leslie E., Owen N. (2001). Perceived Environmental Aesthetics and Convenience and Company Are Associated with Walking for Exercise among Australian Adults. Prev. Med..

[3] Ahn S.H. (2023). Effects of Walking and Outdoor Equipment Exercise on Inflammatory Factors and Metabolic Syndrome indicators in Elderly Women. J. Korean Soc. Sports Sci..

[4] Kim Y.W., Kwon Y.M. (2024). A Meta-Analysis of the Effect of a Walking Exercise Program Applied to Koreans. J. Sport Leis. Stud..

[5] Mohdi A., Mehdi M., Muhammad Z.S., Zohreh A.S., Mehdi A.K. (2018). Evaluating Capability Walkability Audit Tools Assessing Sidewalks. Sustain. Cities Soc..

[6] Frank L.D., Sallis J.F., Conway T.L., Chapman J.E., Saelens B.E., Bachman W. (2007). Many Pathways from Land Use to Health: Associations between Neighborhood Walkability and Active Transportation, Body Mass Index, and Air Quality. J. Am. Plann. Assoc..

[7] Landis B.W., Vattikuti V.R., Ottenberg R.M., McLeod D.S., Guttenplan M. (2001). Modeling the Roadside Walking Environment: Pedestrian Level of Service. Transp. Res. Rec..

[8] Ng H.R., Sossa I., Nam Y.W., Youn J.H. (2022). Machine Learning Approach for Automated Detection of Irregular Walking Surfaces for Walkability Assessment with Wearable Sensor. Sensors.

[9] Ng H.R., Zhong X., Nam Y.W., Youn J.H. (2023). Deep-Learning-Based Approach for Automated Detection of Irregular Walking Surfaces for Walkability Assessment with Wearable Sensor. Appl. Sci..

[10] Zhao G., Cao M., Vos J.D. (2024). Exploring Walking Behaviour Perceived Walkability Older Adults in London. J. Transp. Health.

[11] Ewing R., Handy S. (2009). Measuring the Unmeasurable: Urban Design Qualities Related to Walkability. J. Urban Des..

[12] Leslie E., Coffee N., Frank L., Owen N., Bauman A., Hugo G. (2007). Walkability of Local Communities: Using Geographic Information Systems to Objectively Assess Relevant Environmental Attributes. Health Place.

[13] Menz H.B., Lord S.R., Fitzpatrick R.C. (2003). Acceleration Patterns of the Head and Pelvis when Walking on Level and Irregular Surfaces. Gait Posture.

[14] Xia K., Huang J., Wang H. (2020). LSTM-CNN Architecture for Human Activity Recognition. IEEE Access.

[15] Chen L., Hoey J., Nugent C.D., Cook D.J., Yu Z. (2012). Sensor-based Activity Recognition. IEEE Trans. Syst. Man Cybern. C.

[16] Tsutsumi H., Kondo K., Takenaka K., Hasegawa T. (2023). Sensor-Based Activity Recognition Using Frequency Band Enhancement Filters and Model Ensembles. Sensors.

[17] Mario M.O. (2019). Human Activity Recognition Based Single Sensor Square HV Acceleration Images Convolutional Neural Networks. IEEE Sens. J..

[18] Manca M.M., Pes B., Riboni D. (2022). Exploiting Feature Selection in Human Activity Recognition: Methodological Insights and Empirical Results Using Mobile Sensor Data. IEEE Access.

[19] Jain A., Kanhangad V. (2018). Human Activity Classification Smartphones Using Accelerometer Gyroscope Sensors. IEEE Sens. J..

[20] Gupta P., Dallas T. (2014). Feature Selection Activity Recognition System Using Single Triaxial Accelerometer. IEEE Trans. Biomed. Eng..

[21] Kim H., Ahn C.R., Yang K.H. (2016). A People-centric Sensing Approach Detecting Sidewalk Defects. Adv. Eng. Inf..

[22] Corazza M.V., Mascio P.D., Moretti L. (2016). Managing Sidewalk Pavement Maintenance: A Case Study to Increase Pedestrian Safety. J. Traffic Transp. Eng..

[23] Golshan H.M., Hebb A.O., Hanrahan S.J., Nedrud J., Mahoor M.H. FFT-based Synchronization Approach Recognize Human Behaviors Using STN-LFP Signal. Proceedings of the 2017 IEEE International Conference on Acoustics, Speech and Signal Processing (ICASSP).

[24] Kim H., Ahn C.R., Nam Y. (2019). The influence of built environment features on crowdsourced physiological responses of pedestrians in neighborhoods. Comput. Environ. Urban Syst..

[25] Sousa N., Coutinho-Rodrigues J., Natividade-Jesus E. (2017). Sidewalk Infrastructure Assessment Using a Multicriteria Methodology for Maintenance Planning. J. Infrastruct. Syst..

[26] Dlhaq D., Basfian M.F., Ayuningtyas N.V., Brilianti D.F. (2024). Analysis of Sidewalk Comfort Level Based on Width, Cleanliness, Surface Condition, Lighting, and Availability of Signage and Directional Indicators on Sudibyo Street Sidewalks in Tegal City. J. Sci. Res. Educ. Technol. (JSRET).

[27] Gao W., Qian Y., Chen H., Zhong Z., Zhou M., Aminpour F. (2022). Assessment of Sidewalk Walkability: Integrating Objective and Subjective Measures of Identical Context-Based Sidewalk Features. Sustain. Cities Soc..

[28] Takahashi J., Kobana Y., Tobe Y. Classification of Steps on Road Surface Using Acceleration Signals. Proceedings of the MOBIQUITOUS 2015.

[29] Damaceno R., Ferreira L., Miranda F., Hosseini M., Cesar R. (2024). SideSeeing: A Multimodal Dataset for Sidewalk Accessibility Assessment Integrating IMU, GPS, and video data. arXiv.

[30] Jiang S., Wang H., Fan W., Min C., Zhang X., Ma J. (2025). A Non-Contact Method for Detecting and Evaluating the Non-Motor Use of Sidewalks Based on Three-Dimensional Pavement Morphology Analysis. Sensors.

[31] He Y., Chen Y., Tang L., Chen J., Tang J., Yang X., Su S., Zhao C., Xiao N. (2024). Accuracy Validation of a Wearable IMU-based Gait Analysis in Healthy Female. BMC Sports Sci. Med. Rehabil..

[32] Miyata A., Araki I., Wang T. (2018). Barrier Detection Using Sensor Data from Unimpaired Pedestrians. Human Aspects of IT for the Aged Population.

[33] Kobayashi S., Hasegawa T. (2021). Smartphone-based Estimation Sidewalk Surface Type Via Deep Learning. Sens. Mater..

[34] Pundlik S., Tomasi N., Houston K.E., Kumar A., Shivshanker P., Bowers A.R., Peli E., Guo G. (2024). Gaze Scanning on Mid-Lock Sidewalks by Pedestrians with Homonymous Hemianopia with or without Spatial Neglect. Invest. Ophthalmol. Vis. Sci..

[35] Wang H., Basu A., Durandau G., Sartori M. (2023). Wearable Real-Time Kinetic Measurement Sensor Setup for Human Locomotion. Wearable Technol..

[36] Küderle A., Roth N., Zlatanovic J., Zrenner M., Eskofier B., Kluge F. (2022). The Placement of Foot-Mounted IMU Sensors Does Affect the Accuracy of Spatial Parameters during Regular Walking. PLoS ONE.

[37] MbientLab MetaMotionR, San Francisco, CA, USA. [Online]. https://mbientlab.com/store/metamotionr/.

[38] Thyagarajan K.S. (2018). Introduction to Digital Signal Processing Using MATLAB with Application to Digital Communications.

[39] Ifeachor E., Jervis B.W. (2001). Digital Signal Processing.

[40] Kim P. (2011). Kalman Filter for Beginners: With MATLAB Examples.

[41] Fawcett T. (2006). An Introduction to ROC Analysis. Pattern Recognit. Lett..

